# IUC24433-81 Docetaxel versus olaparib plus abiraterone as first-line treatment in patients with mCRPC: a retrospective single-center analysis

**DOI:** 10.1093/oncolo/oyaf248.039

**Published:** 2025-08-29

**Authors:** M Maffezzoli, L Pedapati, M Hassan, S A Sandulache, J Samuel, F Di Costanzo, G Neola, J Lau, A Maniam, G Banna

**Affiliations:** Portsmouth University Hospitals NHS Trust, Portsmouth, United Kingdom; Portsmouth University Hospitals NHS Trust, Portsmouth, United Kingdom; Portsmouth University Hospitals NHS Trust, Portsmouth, United Kingdom; Portsmouth University Hospitals NHS Trust, Portsmouth, United Kingdom; Portsmouth University Hospitals NHS Trust, Portsmouth, United Kingdom; The Newcastle Upon Tyne Hospitals NHS Foundation Trust, Newcastle, United Kingdom; Federico II University, Naples, Italy; Portsmouth University Hospitals NHS Trust, Portsmouth, United Kingdom; Portsmouth University Hospitals NHS Trust, Portsmouth, United Kingdom; Portsmouth University Hospitals NHS Trust, Portsmouth, United Kingdom

## Abstract

**Background:**

Olaparib and abiraterone (Ola+Abi) has emerged as a novel first-line (1L) treatment option for metastatic castration-resistant prostate cancer (mCRPC), but comparisons with docetaxel are lacking. We retrospectively evaluated outcomes of patients treated with either docetaxel (Doc) or Ola+Abi as 1L therapy for mCRPC.

**Methods:**

This real-world study included patients with mCRPC who received 1L treatment with either Doc or Ola+Abi. Radiographic progression-free survival (rPFS) was assessed using Kaplan–Meier estimates and compared by log-rank test.

**Results:**

Sixty patients were included: 24 (40%) received Doc and 36 (60%) received Ola+Abi. In the hormone-sensitive (HS) setting, 28 patients (77.8%) in the Ola+Abi group had received Doc, while in the Doc group, 12 patients (50%) had received ADT, 11 (45.8%) enzalutamide and 1 (4.2%) apalutamide. Baseline characteristics were well balanced except for the previous HS disease setting (*P *=* *.015), although time to castration resistance was not significantly different (*P *=* *.159) (Table 1).

Median rPFS was significantly longer with the Ola+Abi group (14.5 months, 95%CI 10.2-NR) compared to Doc (8.2 months, 95%CI 3.5-10.9), *P *=* *.004 (Figure 1).

**Conclusions:**

Ola+Abi was associated with improved rPFS compared to Doc as 1L treatment for mCRPC. Despite limitations due to retrospective design, small sample size, and possible differences in characteristics between the 2 groups, these results suggest different clinical outcomes between treatment strategies, which warrant overall survival data.

**Table 1:** Summary of baseline characteristics.

	Doc (*N* = 24)	Ola+Abi (*N* = 36)	Total (*N* = 60)	*P* value
**ECOG PS**				.129
**0**	12.0 (50.0%)	25.0 (69.4%)	37.0 (61.7%)	
**1**	12.0 (50.0%)	11.0 (30.6%)	23.0 (38.3%)	
**Age**				.617
**Mean (SD)**	70.5 (9.3)	69.4 (8.1)	69.8 (8.5)	
**PSA at diagnosis**				.777
**Mean (SD)**	280.0 (555.9)	240.3 (513.1)	256.2 (526.3)	
**Hormone-sensitive setting**				**.015**
**De novo LV**	5.0 (20.8%)	11.0 (30.6%)	16.0 (26.7%)	
**De novo HV**	9.0 (37.5%)	12.0 (33.3%)	21.0 (35.0%)	
**Metachronous LV**	0.0 (0.0%)	9.0 (25.0%)	9.0 (15.0%)	
**Metachronous HV**	2.0 (8.3%)	1.0 (2.8%)	3.0 (5.0%)	
**Non-metastatic**	8.0 (33.3%)	3.0 (8.3%)	11.0 (18.3%)	
**Time to castration resistant**				.159
**Mean (SD)**	21.0 (14.3)	27.4 (18.7)	24.9 (17.3)	
**Range**	5.5-63.0	3.2-88.6	3.2-88.6	

**Figure 1.**
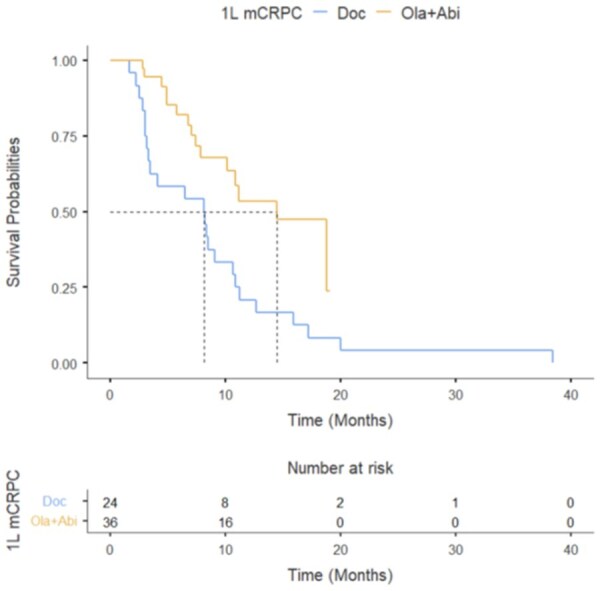
Kaplan–Meier curve for rPFS.

